# Effects of a Seven-Week App-Based Passive Psychoeducation Program on Perceived Stress in Nursing Students—A Randomized Controlled Parallel-Group Study

**DOI:** 10.3390/nursrep16090295

**Published:** 2026-08-24

**Authors:** Elisabeth M. Weiss, Markus Canazei, Siegmund Staggl, Laura Staller, Katharina Kreiger, Bernhard Holzner, Verena Dresen

**Affiliations:** 1 Department of Psychology, University of Innsbruck, 6020 Innsbruck, Austria; elisabeth.weiss@uibk.ac.at (E.M.W.); markus.canazei@uibk.ac.at (M.C.); siegmund.staggl@uibk.ac.at (S.S.); laura.staller@uibk.ac.at (L.S.); katharina.kreiger@student.uibk.ac.at (K.K.); 2 Rheinburg Klinik, Kliniken Valens, 9428 Walzenhausen, Switzerland; 3 Department of General and Social Psychiatry, University Hospital for Psychiatry I, Medical University of Innsbruck, 6020 Innsbruck, Austria; bernhard.holzner@tirol-kliniken.at

**Keywords:** passive psychoeducation, web-based intervention, nursing students, stress

## Abstract

**Background/Objective:** Web-delivered psychoeducational tools offer scalable options for managing elevated stress. Passive psychoeducation that delivers self-guided content without active psychotherapy techniques or homework may be particularly accessible at low costs. The present study examined the acceptability and preliminary effectiveness of a seven-week app-based passive psychoeducational stress-management program in an unselected cohort of nursing students. **Methods:** In this parallel-group randomized controlled trial (preregistered at the German Clinical Trials Register), 154 nursing students were randomized to either a psychoeducation group (*n* = 77) or a waitlist control (*n* = 77). Outcomes were assessed at baseline and post-intervention using the Depression Anxiety Stress Scales–21 (DASS-21). A total of 56 completed the post-test assessment. The intervention group received a new psychoeducational module each week for seven weeks. After each module, participants rated satisfaction and reported adherence and application of content in daily life. The control group received no intervention. **Results:** Compared with the waitlist control, the intervention group showed a statistically significant reduction in perceived stress from pre- to post-test. Depressive symptoms demonstrated a trend toward improvement, whereas anxiety scores did not change significantly. Acceptability ratings were high and participants reported moderate uptake of strategies and perceived relief. **Conclusions:** A brief, app-based passive psychoeducational program appears feasible and acceptable for nursing students and can reduce perceived stress. However, the findings of the current study should be interpreted cautiously given the high attrition rate and the absence of long-term follow-up. Further studies with larger samples and replication under conditions of better retention are warranted.

## 1. Introduction

Nursing students navigate a uniquely demanding educational pathway that combines intensive academic requirements with emotionally charged clinical experiences. Early and repeated exposure to human suffering, death, ethical dilemmas, and high-stakes decision-making occurs in parallel with developmental transitions typical of emerging adulthood, financial pressures, and role strain [1]. The COVID-19 pandemic further amplified these pressures through fear of infection, social isolation, heightened workload, and pervasive uncertainty, thereby increasing the risk of mental disorders [2]. Unsurprisingly, elevated rates of stress, anxiety, and depression are reported in nursing students, consistently exceeding rates observed among age-matched peers [3], with meta-analytic evidence indicating that approximately one-third [4,5] to one half of nursing students [6,7,8] screen positive for clinically significant mental health problems.

Psychological distress not only compromises students’ well-being but is associated with impaired cognitive performance [9], absenteeism, reduced clinical confidence, and attrition from programs [10], which are factors that ultimately affect workforce sustainability and patient care quality [11]. Although higher education institutions have expanded counseling services, many students underutilize formal supports because of perceived stigma [12,13], time constraints, low mental health literacy, and uncertainty about when and how to seek help [14,15]. These realities underscore the need for effective, scalable stress-management strategies that are accessible, acceptable, and feasible within the constraints of nursing curricula and clinical training.

Digital delivery offers a promising route to meet these needs. Internet- and app-based interventions can provide flexible, asynchronous access and microlearning that accommodates variable schedules and placement demands [16]. Within this space, a continuum ranges from higher-intensity, active digital therapeutics, such as guided internet-delivered cognitive behavioral therapy or mindfulness-based programs that require skills practice and homework, to lower-intensity approaches that focus primarily on psychoeducation. An active stress-management program generally produces small but significant reductions in stress as documented in meta-analyses and reviews [17,18,19], and related outcomes among university students (e.g., [20]), but can be resource intensive, time-consuming, and susceptible to adherence barriers.

In contrast, passive psychoeducation, defined here as the structured, evidence-informed provision of information about mental health, is a low-cost, time-saving/schedule-flexible, and accessible approach that delivers information via apps, websites, or print materials without incorporating active psychotherapy components or requiring at-home exercises. Psychoeducation is an established component of prevention and early intervention across diverse populations, including nursing students [21,22,23].

Core elements typically include information about the normalization of stress responses; education about common symptoms, trajectories, and risk/protective factors; basic cognitive-behavioral and problem-solving strategies, information about stress management and healthy lifestyle, and clear pathways to support services [3]. It rests on the premise that receiving appropriate information can influence individuals and their behavior by motivating them to apply the acquired knowledge and adjust their actions accordingly [21]. Passive psychoeducation can strengthen adaptive coping and emotion regulation [20], enhance knowledge and self-efficacy, reduce perceived stigma, and increase intentions to seek help [24], thereby improving well-being and in some cases, alleviating symptoms of stress and anxiety [25]. For nursing students, who must rapidly develop professional resilience alongside clinical competence, low-intensity digital psychoeducation could serve as a first-line or stepped-care entry point that minimizes burden while promoting early skill uptake and help-seeking.

Despite the growing availability of digital mental health tools, key evidence gaps persist regarding low-intensity, information-only approaches. While many established interventions have been adapted for online delivery and targeted conditions, including anxiety, depression, and stress [26,27], and meta-analyses support the overall effectiveness of internet interventions for mental disorders (e.g., [28]), most trials test active, skill-based programs with guided practice. Preventive approaches targeting people with subclinical or less severe symptoms are gaining attention [29,30], yet the specific effectiveness and acceptability of passive psychoeducation remain under-examined, and evidence specific to nursing students, who face distinct clinical and scheduling demands, is particularly sparse.

In a previous study, we demonstrated that a seven-week, app-based passive psychoeducation program produced significant improvements in selected adaptive emotion regulation and coping skills among university students with subthreshold psychopathology [20] and we derived design principles salient for nursing curricula: brevity and modularity to align with crowded timetables, integration with clinical learning to enhance applicability, and explicit signposting to institutional and community resources.

Building on this rationale, the present randomized controlled trial evaluates the acceptability and effectiveness of a seven-week app-based passive psychoeducational stress-management program in an unselected cohort of nursing students, a group that exhibited significantly higher levels of depression, anxiety, and stress than the university sample in our earlier study [20]. By focusing on a low-intensity, scalable intervention, this trial addresses the current evidence gap regarding whether passive psychoeducation can meaningfully reduce distress and be perceived as useful and applicable in everyday academic and clinical contexts. Given that the psychoeducation program was explicitly designed as a stress-management intervention, we pre-specified perceived stress as the primary outcome and depressive and anxiety symptoms as secondary outcomes. Drawing on evidence that brief stress-management approaches produce the most immediate and reliable reductions in perceived stress, we hypothesized greater pre–post reductions in stress for the intervention group relative to a waitlist control, with smaller or more variable effects on depressive symptoms and minimal short-term change in anxiety. Consistent with our feasibility aims, we also expected high acceptability of the program and moderate uptake of its strategies, accompanied by perceived relief.

## 2. Materials and Methods

**Figure 1:**
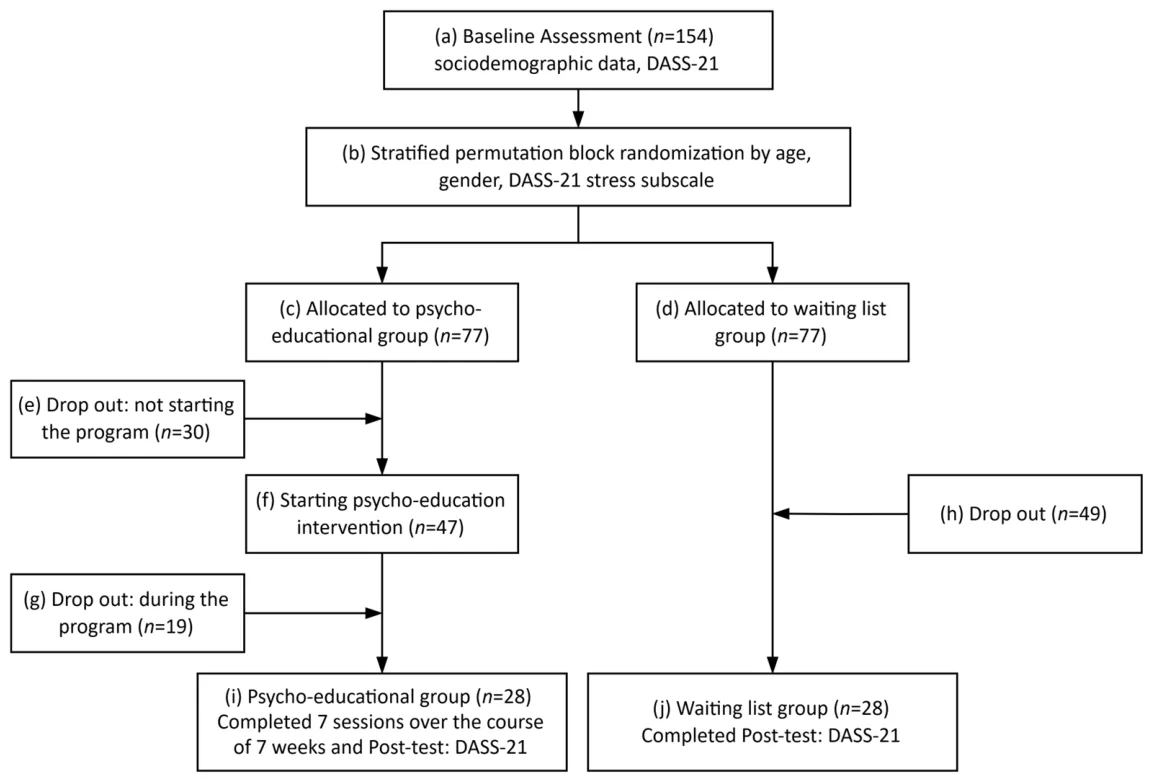
Flow diagram of the present study: (a) 154 nursing students completed baseline assessment (pre-test), including sociodemographic data and Depression Anxiety Stress Scales (DASS-21); (b) after the pre-test a stratified permuted-block randomization was applied, and allocation was balanced on the DASS-21 Stress subscale score, age, and gender of nursing students; (c) 77 nursing students were allocated to the psychoeducation arm; (d) 77 nursing students were allocated to the waiting list control arm; (e) of those randomized to psychoeducation, 30 participants did not start the program; (f) in the psychoeducation group, 47 participants initiated the program; (g) in the psychoeducation group, 19 participants were excluded because of incomplete data or drop out during the seven weeks; (h) 49 drop outs in the waiting list control group; (i) 28 participants completed both the seven-week intervention and the DASS-21 at the seven-week follow-up; (j) in the waiting list control group, 28 participants completed the DASS-21 at the seven-week follow-up.

Nursing students in Austria and Germany were recruited via invitation letters disseminated by the heads of their schools. Data were collected from March 2022 until April 2023. In total, 154 nursing students (116 women, 38 men) took part in a screening study comparing the mental health impact of COVID-19 between nursing and non-nursing students [3]. The present work reports on a seven-week, parallel-group randomized controlled trial nested within that screening (preregistered at the German Clinical Trials Register). Participants completed assessments at baseline and post-intervention using the Depression Anxiety Stress Scales (DASS-21; Leibniz-Institut für Psychologie (ZPID), Open Test Archive: Trier, Germany [31]). Following the baseline assessment, stratified permuted-block randomization was applied; allocation was balanced on the DASS-21 Stress subscale score, age, and gender of nursing students, yielding a psychoeducation arm (*n* = 77) and a waitlist control arm (*n* = 77). Of those randomized to psychoeducation, 47 initiated the program and 28 completed both the seven-week intervention and the post-test. In the waitlist control group, 28 participants completed the DASS-21 at the seven-week follow-up. Participation was voluntary, written informed consent was obtained from all participants, and procedures adhered to the Declaration of Helsinki (1964) with approval from the local ethics committee. An overview of study flow and measures is provided in Figure 1.

### 2.1. Procedure

Participants assigned to psychoeducation received a new module each Monday for seven consecutive weeks, accompanied by a smartphone push notification. The program was delivered via the CHES platform [32] and comprised seven modules (see [20], for detailed content): (1) The first module provides an overview of stress responses and the physiological and psychological consequences of prolonged stress, underscores the importance of recovery processes, and offers a brief introduction to self-management strategies. (2) The second module focuses on daily routine management, emphasizing time management and task prioritization through strategies such as to-do lists, mapping performance peaks and troughs, and creating realistic schedules with brief pre- and post-task breaks. (3) The third module examines emotion regulation and problem solving by contrasting maladaptive strategies (e.g., suppression, avoidance, rumination) with adaptive approaches (e.g., emotion recognition, acceptance, reappraisal, positive refocusing) and introducing a six-step problem-solving model. (4) The fourth module addresses sleep, emphasizing healthy sleep–wake patterns and sleep hygiene, including consistent bed/wake times, avoiding naps, engaging in daytime physical activity, and optimizing the sleep environment. (5) The fifth module outlines the health benefits of pleasurable leisure activities, catalogs activity types, and provides guidance for effective planning. (6) The sixth module emphasizes the role of physical activity in physical and mental health, detailing stress-reducing and strength-/mobility-enhancing exercises and practical strategies for integrating regular activity into daily life. (7) The final module on nutrition synthesizes evidence-based dietary recommendations, including adequate hydration, and provides tailored guidance for vegetarian and vegan diets alongside resources for meal planning. Each module required approximately 10–15 min to complete. Participants could revisit the full module content on the website as often and as long as they wished. Usage was tracked via back-end log-ins, and text message reminders were issued to encourage timely engagement and completion. At the final visit, participants evaluated the intervention and provided open-ended feedback on desired features for future iterations. Satisfaction with content, pace, and design was rated on a 5-point Likert scale.

Participants in the waitlist control condition received no intervention but were informed that full access to the psychoeducation materials would be granted after the post-test assessment.

### 2.2. Measurements

#### 2.2.1. Depression Anxiety Stress Scale–21 (DASS-21, Leibniz-Institut Für Psychologie (ZPID), Open Test Archive: Trier, Germany [31])

Mental health was assessed using the German version of the 21-item Depression Anxiety Stress Scales (DASS-21, APA PsycTests: Washington, DC, USA [33]; German version [31]). Participants rated how much each statement applied to them over the past week on a 4-point Likert scale (0 = “did not apply to me at all”, 3 = “applied to me very much, or most of the time”). The measure yields three subscales: depression, anxiety, and stress, each comprising seven items. Subscale scores were computed as the sum of the relevant items (ranging from 0 to 21), where higher scores indicate greater symptom severity. The three subscales have good psychometric properties, including internal consistency and validity [34]. The convergent and discriminant validity of the DASS-21 are sufficient [34]. In the current study, internal consistency was acceptable to good for all DASS-21 subscales (Cronbach’s α: depression = 0.87; anxiety = 0.77; stress = 0.83).

#### 2.2.2. Module-Specific Satisfaction and Adherence Questionnaire

Following each module, participants evaluated their satisfaction with the content using two items: (1) “How helpful did you find this week’s informational material?”. Responses were recorded on a 4-point Likert scale (1 = not helpful; 2 = minimally helpful; 3 = helpful; 4 = very helpful). (2) “How well informed did you feel about the topic?” Again, responses were recorded on a 4-point Likert scale ranging from 1 = very bad, 2 = moderate, 3 = good, to 4 = very good.

To assess engagement and adherence to the app-based passive psychoeducational stress-management program, participants answered two additional items: (1) “Were you able to apply the knowledge from the psychoeducation in everyday life during the past week?” and (2) “Did you feel relieved by using the psychoeducation?” These items were rated on a 4-point Likert scale from 1 (never), 2 (rarely; on 1–2 days), 3 (frequently; on 3–4 days), to 4 (regularly; more than five days per week).

For statistical analyses, item scores were averaged across the seven modules. Additionally, participants rated the importance of each of the seven module topics (e.g., “Is the topic of stress and recovery of high importance to you?” (Yes/No). Finally, participants responded to open-ended prompts soliciting suggestions for additional features to be incorporated in future iterations of the program.

### 2.3. Sample Size and Statistical Power

No a priori sample size calculation was performed during the planning phase of this trial. The achievable sample size was constrained by feasibility: the trial was nested within a larger study [3], and enrollment was limited to the number of eligible nursing students available at the collaborating institutions during the recruitment period. However, we have performed a post hoc sensitivity (detectable-effect) analysis with G*Power (Version 3.1) based on the realized sample size. With the randomized sample (*N* = 154, *n* = 77 per arm), α = 0.05 (two-tailed) and 80% power, the smallest detectable between-group effect was *d* = 0.45 for an unadjusted comparison and *d* = 0.33 for an analysis adjusting the corresponding baseline score (*f* = 0.23, ηp^2^ = 0.049). For the subsample with complete post-intervention data (*N* = 56), the corresponding values were *d* = 0.76 and *d* = 0.55 (*f* = 0.38, ηp^2^ = 0.127). Detecting a medium-sized effect (*d* = 0.50) with 80% power would have required 64 participants per arm (*N* = 128), or a total of *N* = 68 with baseline adjustment. The randomized sample therefore exceeded the requirement for medium effects, whereas the number of completers did not.

### 2.4. Statistical Analyses

All statistical analyses were performed in SPSS 26.

Data analysis included participants with complete baseline and second measurement data (PP analysis). Descriptive statistics are presented as mean (M) and standard deviation (SD). To compare demographic variables between the two groups, either t-tests, chi-square tests or Fisher’s exact tests were performed. Multivariate analyses of variance (MANOVA) were performed to examine baseline group differences in the DASS-21 scales, using the scores of each scale as dependent variables.

A repeated measure (multivariate) analysis of variance (rMANOVA) was conducted to examine changes in the DASS-21 scales over time, using the between-subjects factor “group” (psychoeducation vs. waiting group) and the within-subjects factor “time” (pre-test vs. post-test assessment). Significant group x time interactions were examined by pairwise post-hoc comparisons. We tested for homogeneity of variances in the between-subjects factor using Box’s M-test. Furthermore, we examined skewness and kurtosis to test for deviations from the normal distribution in the dependent variables. Unless otherwise stated in Section 3, we confirmed that all these assumptions were met. For significant effects, we report partial eta-squared effect sizes (ηp^2^). All analyses were conducted with a significance level of 0.05 (two-tailed).

Because of the substantial attrition, all outcome analyses were repeated as intention-to-treat analyses including all 154 randomized participants. Missing post-intervention DASS-21 scores were addressed by multiple imputation using chained equations with predictive mean matching (five donors), generating *m* = 100 imputed datasets with 10 iterations each. The imputation model comprised group allocation, all three baseline DASS-21 subscale-scores, sex, age, and the remaining post-intervention DASS-21 subscales. Predictive mean matching ensured that imputed values remained within the observed range of the scales.

In each imputed dataset, group differences were tested by analysis of covariance of the post-intervention score, with group as a fixed factor and the corresponding baseline score as a covariate; estimates were combined using Rubin’s rules with Barnard–Rubin degrees of freedom. Robustness was assessed in five sensitivity analyses: complete-case analysis, imputation performed separately within each trial arm, base-line observation carried forward, control-based imputation, and a delta-adjusted pattern-mixture analysis that progressively penalized the imputed values of the psychoeducation group. Multiple imputation and the associated analyses were performed in Python 3.13 (pandas, statsmodels).

## 3. Results

### 3.1. Sample Characteristics

Demographic details of the screening sample are reported in Dresen et al. [3]. A total of 47 female and nine male participants completed the seven-week, parallel-group randomized controlled trial. The age range of the sample was between 17 and 60 years (*M* + *SD* = 28.64 + 11.27). Sociodemographic and study-related characteristics by group are summarized in Table 1.

No significant baseline differences were observed between the psychoeducation and waitlist groups for age, gender, working hours, current or past mental illness (Fisher’s exact test), physical illness, or psychological/psychotherapeutic/psychiatric treatment (all *p*’s > 0.05).

A MANOVA on the DASS-21 subscales at baseline indicated no significant between-group differences (*F*(3, 52) = 0.578, *p* = 0.632, ηp^2^ = 0.032). Descriptive statistics for pre-test variables are presented alongside post-test outcomes in Table 2.

### 3.2. Mental Health Outcomes

**Figure 2:**
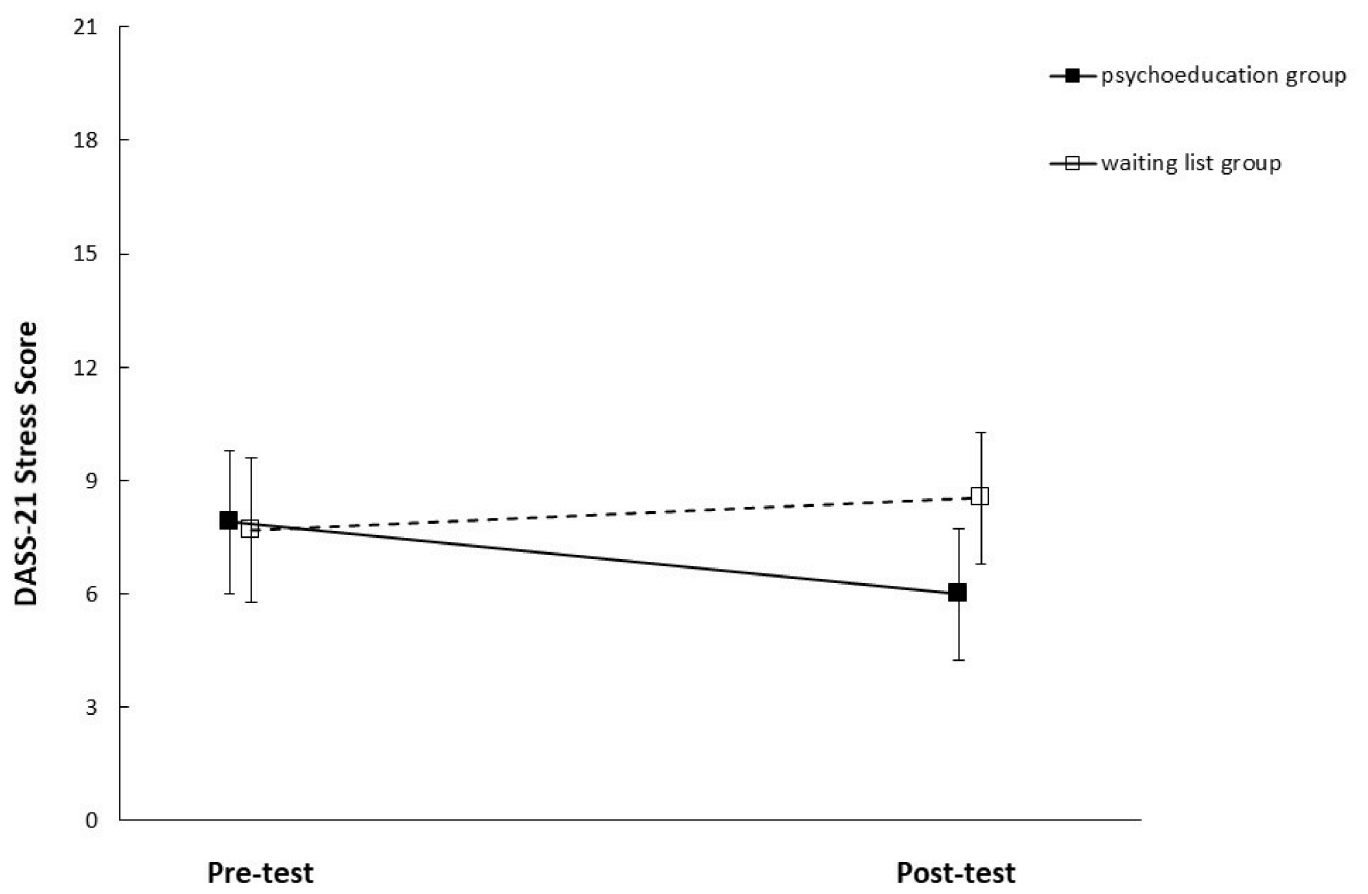
Changes in the DASS-21 stress subscales from pre- to post-test. Note: the figure shows means and 95% confidence intervals of the means.

**Figure 3:**
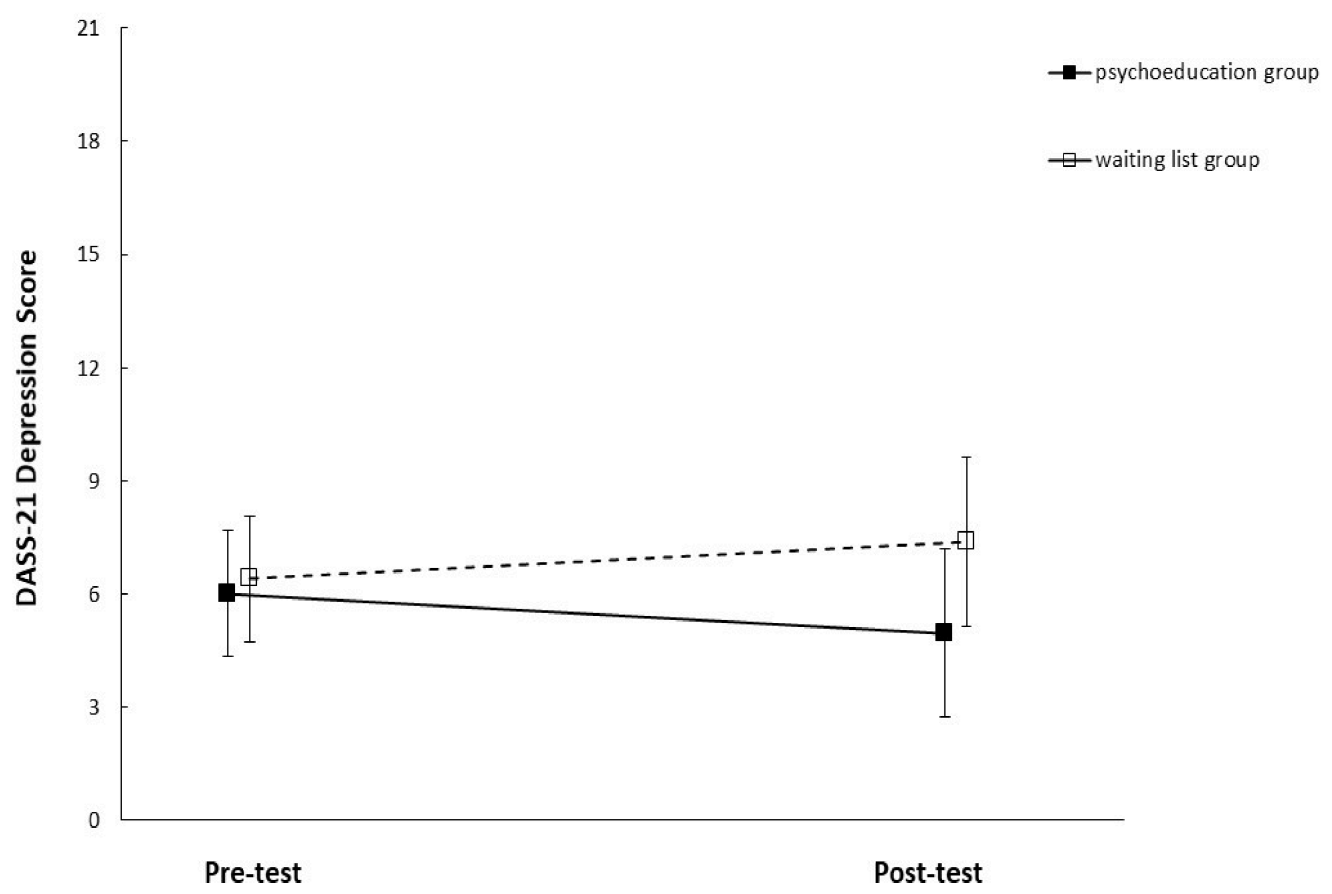
Changes in the DASS-21 depression subscales from pre- to post-test. Note: the figure shows means and 95% confidence intervals of the means.

A rMANOVA on DASS-21 depression, anxiety, and stress subscales revealed a significant group × time interaction (*F*(3, 52) = 2.91, *p* = 0.043, ηp^2^ = 0.144). No significant main effects were found for group (*F*(3, 52) = 0.862, *p* = 0.467, ηp^2^ = 0.047) and time (*F*(3, 52) = 0.565, *p* = 0.64, ηp^2^ = 0.032). Follow-up tests indicated a significant pre–to-post decrease in the DASS-21 stress subscale for the psychoeducation group relative to the waiting list control group (*F*(1, 54) = 8.386, *p* = 0.005, ηp^2^ = 0.134), see Figure 2. In the psychoeducation group a trend toward significant improvement was observed for depression relative to the waiting list control group (*F*(1, 54) = 3.622, *p* = 0.062, ηp^2^ = 0.063), see Figure 3, whereas anxiety scores did not change significantly (*F*(1, 54) = 2.483, *p* = 0.121, ηp^2^ = 0.044).

### 3.3. Intention-to-Treat Analysis

Baseline characteristics of completers and non-completers and sensitivity analyses for the intention-to-treat analysis of the DASS-21 subscales are presented in the Supplementary Material (Tables S1 and S2).

Post-intervention data were missing for 98 of the 154 randomized participants (63.6%), with identical attrition in both arms (49 of 77). Little’s test was consistent with data missing completely at random (χ^2^(3) = 3.56, *p* = 0.313). A logistic regression predicting dropout from group allocation, all three baseline DASS-21 subscale scores, age, and sex was non-significant overall (χ^2^(6) = 7.55, *p* = 0.273). Sex was the only individual predictor (*b* = −0.92, *p* = 0.036), with women being less likely to drop out. Completers and non-completers did not differ on any baseline measure (all *p* > 0.12, all |*d*| ≤ 0.26, see Table S1).

In the intention-to-treat analysis including all 154 randomized participants, the psychoeducation group showed a significantly greater reduction in perceived stress than the waitlist control group (baseline-adjusted mean difference *b* = −2.48, SE = 0.88, 95% CI [−4.24, −0.72], *t*(50.7) = −2.84, *p* = 0.007, *d* = −0.53). Differences on the depression subscale (*b* = −1.53, 95% CI [−3.63, 0.57], *p* = 0.150, *d* = −0.25) and the anxiety subscale (*b* = −0.94, 95% CI [−2.83, 0.94], *p* = 0.318, *d* = −0.20) were not statistically significant, although both point estimates favored the intervention. Results are summarized in Table 3.

The stress effect proved robust across sensitivity analyses (Table S2). It remained significant when imputation was performed separately within each trial arm (*b* = −2.55, *p* = 0.010) and under the conservative baseline-observation-carried-forward approach (*b* = −0.96, *p* = 0.010). In the delta-adjusted pattern-mixture analysis, the effect remained significant as long as the imputed post-intervention stress scores of dropouts in the psychoeducation group were penalized by less than 1.1 points. Only under the extreme control-based assumption that all dropouts in the psychoeducation group would have responded exactly like waitlist participants did the effect lose statistical significance (*b* = −0.80, *p* = 0.315), while retaining its direction.

### 3.4. Satisfaction, Engagement, and Adherence

**Figure 4:**
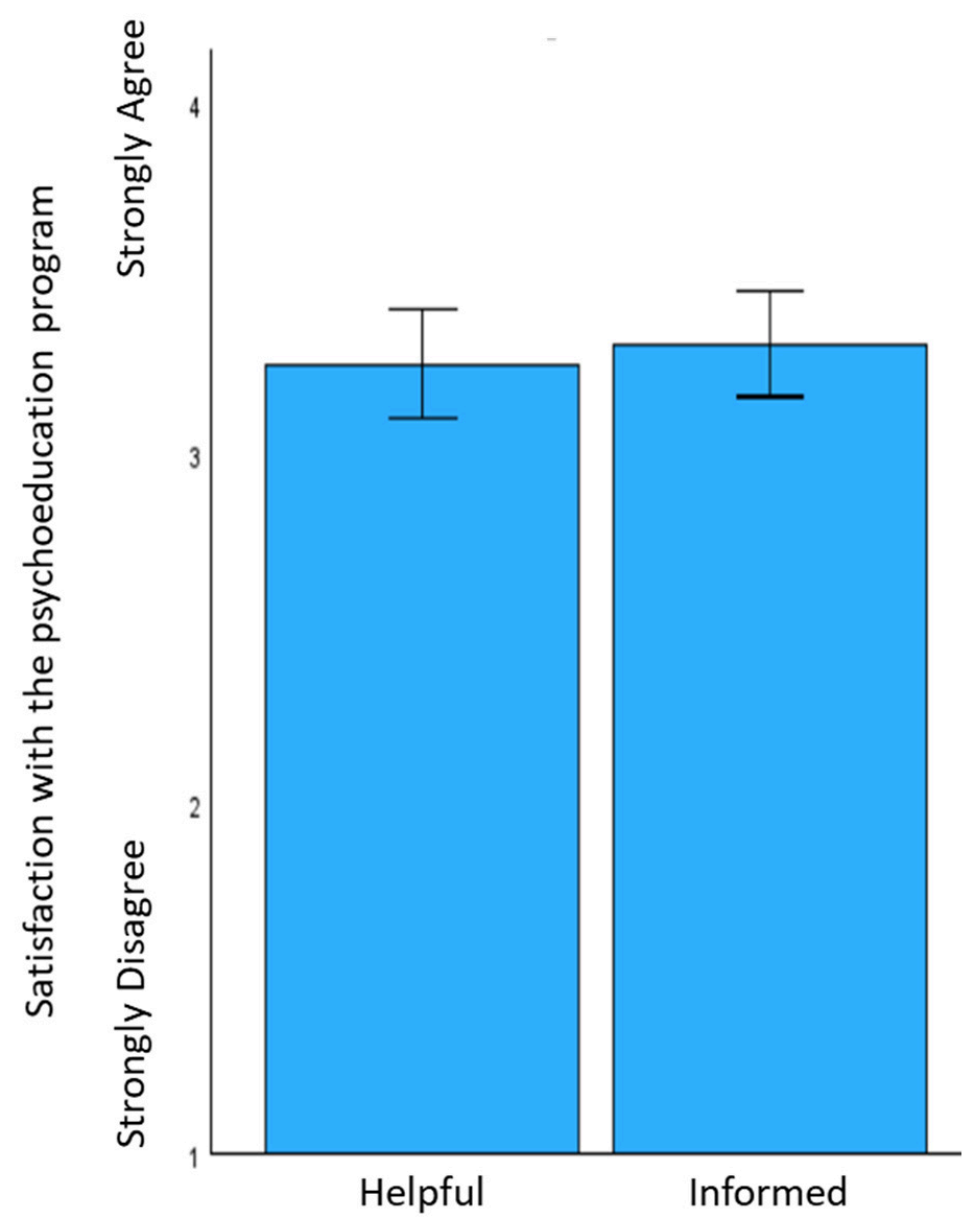
Satisfaction with the psychoeducation program. Note: the figure shows means and 95% confidence intervals of the means.

**Figure 5:**
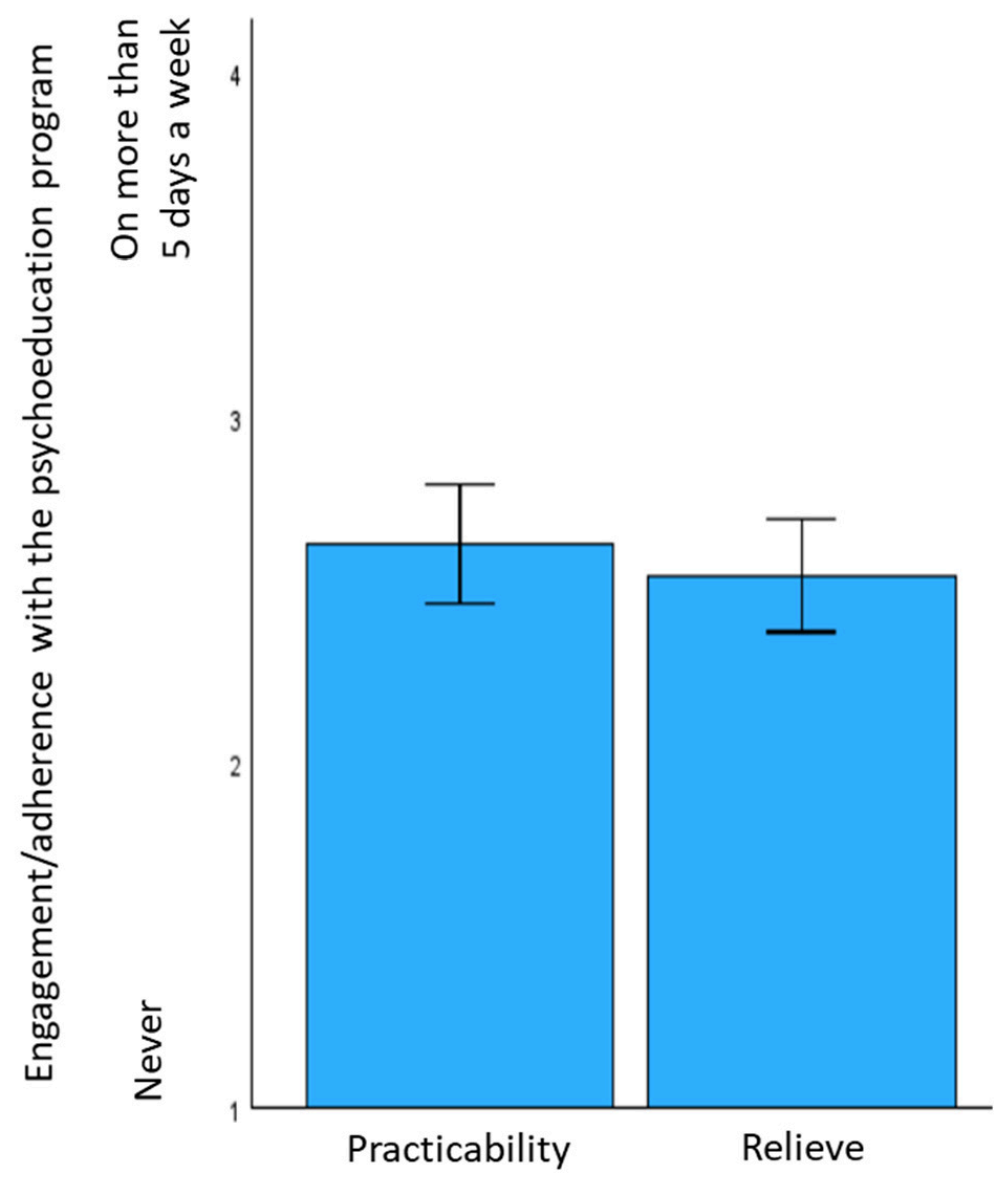
Engagement and adherence with the psychoeducation program. Note: the figure shows means and 95% confidence intervals of the means.

Satisfaction with the psychoeducation program was high across both the usefulness and informational quality scale (helpful: M = 3.26, SD = 0.36; informed: M = 3.33, SD = 0.36). Participants reported applying the knowledge of the modules on approximately half of the days per week (M = 2.64, SD = 0.42) and experiencing relief attributable to the psychoeducation program (M = 2.56, SD = 0.39). Mean ratings of satisfaction and engagement/adherence, including mean scores with 95% confidence intervals, are presented in Figure 4 and Figure 5.

### 3.5. Qualitative Feedback

Seventy-eight percent of participants endorsed stress and regeneration as an important topic, followed by the topics emotion regulation and problem solving (64%), sleep (53%), daily routine (35%), physical exercise (32%), leisure activities (18%), and nutrition (14%).

For future iterations of the psychoeducation program, the nursing students recommended: (1) integrating audiovisual materials to complement textual content and enhance engagement, (2) incorporating illustrative examples grounded in realistic everyday contexts to facilitate transfer to practice, and (3) extending practice periods beyond one week to support sustained skill acquisition and consolidation.

## 4. Discussion

This randomized controlled trial evaluated the acceptability and effectiveness of a seven-week, app-based passive psychoeducational stress-management program for nursing students. Consistent with our hypotheses, the intervention produced a statistically significant reduction in perceived stress relative to a waitlist control. Depressive symptoms showed a trend toward improvement, whereas anxiety did not change significantly. Acceptability ratings were high, and participants reported moderate application of learned content in daily life as well as perceived relief, indicating that a brief, app-delivered, passive psychoeducational format is both feasible and acceptable in this population.

The primary outcome (i.e., a significant reduction in stress) achieved statistical significance with a moderate effect size. This effect is comparatively strong for a low-intensity, unguided psychoeducational intervention in student samples—exceeding average effects typically reported for unguided passive psychoeducation (e.g., [25])—and closer to effects reported for guided or more active, skills-based programs (e.g., [35]). Moreover, the predominance of women in nursing education may align with findings from Van Daele et al. [19] who conclude that short lasting psychoeducational interventions were most effective for women, potentially contributing to the observed magnitude.

The primary stress reduction aligns with the core targets of the intervention, which emphasized psychoeducation on stress physiology, daily structure, sleep, and adaptive coping strategies. Previous meta-analyses have shown that psychoeducation programs for stress management with active interventions (e.g., mindfulness-based interventions or cognitive behavioral techniques), delivered either in face-to-face group settings or internet-based formats, are effective in reducing stress and related symptoms in student samples (e.g., meta-analyses and reviews by [17,19]). Several mechanisms likely underlie the observed stress reduction. Psychoeducation can enhance mental health literacy and perceived control, reduce stigma, and strengthen self-efficacy [24,25], which in turn facilitate adaptive coping and emotion regulation and can reduce stress and related symptoms [21,36]. Content on stress, emotion regulation and sleep—topics participants rated as highly salient—likely supported behavioral regulation known to buffer stress reactivity and improve emotion regulation [36]. Participants reported applying program content on approximately half of the days each week and experiencing relief, suggesting that accessible, structured information can motivate behavior change without formal assignments or coaching [21,25]. In our previous study with university students, a similar seven-week passive psychoeducation program improved adaptive emotion regulation and coping skills, suggesting that knowledge acquisition and normalization mechanisms can translate into meaningful stress reductions [20]. Notably, the program reduced stress in a cohort with comparatively higher baseline symptom burden than our earlier student sample [20], indicating potential utility even when distress levels are substantial.

By contrast, anxiety did not significantly improve, and depression showed only a trend for improvement. Passive psychoeducation primarily targets mental health literacy, normalization, and general self-management strategies. While these elements can reshape stress appraisals and promote adaptive routines (e.g., sleep hygiene, time management), they may be insufficient to produce short-term change in anxiety without active components such as guided cognitive restructuring, exposure, or mindfulness practice [16,36]. Importantly, our program was not designed to address anxiety or depressive symptoms in the way a dedicated psychotherapeutic intervention would focus on. Previous studies indicate that unguided or passive formats tend to yield smaller effects than guided or therapist-assisted programs, particularly for anxiety and depression [16,28,29], albeit at lower resource cost. Moreover, anxiety among nursing students is often tied to evaluative pressures (e.g., examinations, clinical assessments) and situational uncertainty [1]. The brief, non-guided modules may not have provided sufficient depth or contextualization on certain symptoms of anxiety and depression to alter these patterns within seven weeks. Measurement timing may also matter: stress, which is more volatile and context-sensitive, may respond earlier to psychoeducation, whereas downstream changes in anxiety and depressive symptoms could require longer exposure or more active techniques [28,36].

Taken together, these findings suggest that a low-intensity, scalable option can serve as a first-line offering to reduce stress and may operate as a gateway to more intensive supports for nursing students as well as students with persistent or more severe symptoms [16,29,37]. In stepped-care terms, the current effect size, while modest at the individual level, may be clinically meaningful at the population level given the potential reach, low cost, and ease of integration into existing curricula. However, given the high attrition rate and the lack of long-term follow-up, the findings should be interpreted with caution. Larger studies and replications under better retention are warranted.

### 4.1. Satisfaction, Relevance, and Design Implications

Satisfaction indicators were strong across modules, with high ratings for usefulness and informational quality, indicating that the program’s brevity, modularity, and asynchronous delivery mode fit the constraints of nursing education, marked by crowded curricula and irregular clinical schedules [1]. Topic salience clustered around stress, emotion regulation, and sleep, which are domains consistently identified in nursing education as both high-need and modifiable [1,2]. Open-ended feedback highlighted three priorities for optimization: (i) integrating audiovisual materials to increase engagement and accessibility; (ii) embedding realistic, profession-relevant examples to facilitate transfer to clinical and academic contexts; and (iii) extending practice periods beyond one week to support consolidation. These recommendations align with best practices in digital learning and behavior change, including multimodal content, contextualized scenarios, and distributed practice for durable learning [16,29].

Embedding psychoeducation within existing courses or clinical seminars, synchronizing delivery with high-stress periods (e.g., prior to examinations or new clinical rotations), and offering “just-in-time” boosters may enhance uptake and impact [16,29].

Institutions could integrate such psychoeducation into stepped-care frameworks: universal access to passive psychoeducation and resource navigation; escalation to guided digital programs or brief group workshops for students with ongoing distress; and referral to clinical services for those screening positive for mental disorders [16,29,37]. This approach leverages scalability while maintaining pathways to higher-intensity care, potentially improving reach and efficiency of campus mental health provision [37].

### 4.2. Future Directions

Building on these findings, future work should implement longer follow-ups to test durability beyond the immediate post-intervention period and to examine whether early stress reductions translate into downstream gains in academic and clinical functioning, attendance, and well-being. Where feasible, inclusion of objective indicators (e.g., actigraphy-derived sleep, passive smartphone sensing, or brief physiological markers) could help triangulate self-reported outcomes.

A second priority is to iteratively enhance the intervention’s active ingredients while preserving efficiency. Adaptive variants that tailor brief skill practice (e.g., micro-CBT, mindfulness exercises, or graded exposure for evaluative stress) to baseline profiles or emergent needs may better address anxiety and low mood without requiring full therapist guidance. Just-in-time delivery, e.g., synchronizing content with predictable stress peaks such as examinations or new clinical rotations, and strategic booster sessions may improve uptake and maintenance. In parallel, co-design with diverse student groups can inform accessibility features (e.g., audiovisual materials, multilingual options) and ensure usability across devices and settings.

Finally, implementation-focused research should evaluate real-world integration within curricula and stepped-care pathways, including reach, adherence, and cost-effectiveness across cohorts and sites. Testing dose–response relationships and moderators (e.g., baseline distress, sleep problems, prior help-seeking) will clarify for whom, when, and under what conditions passive psychoeducation is most beneficial, thereby guiding rational escalation to guided digital programs or clinical services when needed.

### 4.3. Limitations

Several limitations should be acknowledged. First, in contrast to our previous study in students [20], attrition from randomization to post-test was substantial, with only 36,4% of the intervention group (*n* = 28/77) completing the study. The high dropout rate is consistent with some unguided digital interventions but significantly reduces power and introduces a high risk of selection bias, as completers may possess higher baseline motivation or lower functional impairment than dropouts. One reason for this difference might be that bachelor students of psychology in our previous study [20] received course credit. The high attrition rate also indicates that while passive app-based delivery is structurally accessible, it lacks the interpersonal or institutional accountability required to sustain engagement in a highly strained student population. Future trials should incorporate proactive engagement strategies (e.g., structured onboarding, motivational prompts, or gamified incentives). In the present trial, we adhered to randomized assignment and complemented per-protocol analyses with an intention-to-treat analysis using multiple imputation under a prespecified missing-at-random (MAR) assumption; this analysis reproduced the reduction in perceived stress. Dropout was similar across arms and unrelated to baseline symptom levels, which is consistent with MAR. Nonetheless, the substantial proportion of post intervention missing data may attenuate the realized benefits of randomization and introduces additional uncertainty. Because the MAR assumption cannot be verified empirically, the findings should be interpreted cautiously and ideally replicated under conditions of improved retention. The final analytical sample size was modest, potentially underpowering detection of small effects for anxiety and depression and precluding subgroup analyses (e.g., by gender or baseline severity). Although the gender distribution reflects the nursing student population, it restricts evaluation of gender-specific effects. Unmeasured contextual factors (e.g., concurrent stressors, clinical placement intensity, or external supports) may also have influenced outcomes [1,2].

Second, the waitlist control limits causal inference regarding specific intervention effects versus expectancy or self-monitoring. An active control (e.g., neutral health education) would better isolate psychoeducational content effects [36].

Third, outcomes relied on self-report with immediate post-intervention assessment. The absence of objective or behavioral indices (e.g., actigraphy for sleep, academic or clinical performance) together with the lack of follow-up constrain conclusions about durability and functional impact. Longer-term follow-up with multimethod endpoints is needed [16,29].

Fourth, despite tracking log-ins, we did not examine dose–response associations or fine-grained engagement patterns that might identify active ingredients or optimal dosing. Incorporating detailed usage analytics and ecological momentary assessments could clarify mechanisms [16].

Fifth, there is an ongoing debate regarding the long-term effectiveness and sustainability of these programs (e.g., [38,39]. To assess durability, behavioral, academic/clinical, and physiological endpoints with 3–12 months follow-ups should be incorporated [16,29].

Sixth, program satisfaction and adherence were assessed with the same four items used in our prior study of healthy university students [20]. Consistent with our previous study in healthy university students [20], however these items have not undergone formal psychometric validation.

Seventh, no a priori sample size/power calculations were performed because the sample was constrained by feasible recruitment within the larger study. A post hoc power/sensitivity analysis using G*Power (3.1) indicated that, given the realized sample, the study would have nominal power to detect effects above a certain magnitude for the primary outcome. However, such post hoc assessments are inherently limited and, together with the substantial post-intervention missingness, likely overstate the effective power. We therefore avoid strong claims about power and acknowledge that some analyses, particularly for smaller effects, may be underpowered and that estimate precision may be reduced.

Finally, the study was conducted in Austria and Germany during a period still influenced by pandemic-related disruptions. Cultural, institutional, and temporal factors may limit generalizability to other settings or post-pandemic cohorts [2]. Replication across diverse educational systems and regions is warranted.

## 5. Conclusions

In an RCT with nursing students, a brief, app-based passive psychoeducational program yielded a significant reduction in perceived stress, high acceptability, and moderate self-reported application of content. Improvements in depressive symptoms trended toward significance, whereas anxiety did not change significantly over seven weeks. These findings indicate that low-intensity digital psychoeducation can serve as a feasible, scalable component of mental health support within nursing education, particularly for stress reduction. However, the high attrition observed underscores that passive delivery alone may not engage the majority of students who initiate the program.

To maximize impact, future iterations should incorporate audiovisual materials and light interactivity (e.g., quizzes, brief exercises) and embed realistic nursing scenarios to contextualize emotion regulation and exam-related anxiety. Additionally, to extend and personalize practice, optional extended practice tracks, adaptive pacing, and “just-in-time” boosters aligned with academic or clinical stress peaks should be offered.

## Figures and Tables

**Table 1:** Sociodemographic and study-related characteristics by group.

	Psychoeducation Group *n* = 28	Waitlist Control Group *n* = 28	Total *n* = 56	Test Statistic
Age [years]: mean (SD)	28 (11.01)	29.29 (3.59)	28.64 (11.27)	*t*(56) = −0.424, *p* = 0.67
Sex				Fisher’s exact test
Female: *n* (%)	25 (89%)	22 (79%)	47 (84%)	(Monte Carlo),
Male: *n* (%)	3 (11%)	6 (21%)	9 (16%)	χ^2^(1) = 1.19, *p* = 0.47
Working-hours/week				
10–19 h	2 (7%)	1 (4%)	3 (5%)	Fisher’s exact test
20–29 h	2 (7%)	2 (7%)	4 (7%)	(Monte Carlo),
>30 h	24 (86%)	25 (89%)	49 (88%)	χ^2^(2) = 0.557, *p* = 0.395
Children				Fisher’s exact test
Yes	9 (32%)	7 (25%)	16 (29%)	(Monte Carlo),
No	19 (68%)	21 (75%)	40 (71%)	χ^2^(1) = 0.359, *p* = 0.77
Mental illness				Fisher’s exact test
Yes	2 (7%)	4 (14%)	6 (11%)	(Monte Carlo),
No	26 (93%)	24 (86%)	50 (89%)	χ^2^(1) = 0.747, *p* = 0.335
Physical illness				Fisher’s exact test
Yes	6 (21%)	9 (32%)	15 (27%)	(Monte Carlo),
No	22 (79%)	19 (68%)	41 (73%)	χ^2^(1) = 0.820, *p* = 0.274
Psychological/Psychotherapeutic/psychiatric treatment				
Current	3 (11%)	4 (14%)	7 (13%)	Fisher’s exact test
Past	6 (21%)	13 (46%)	19 (34%)	(Monte Carlo),
No	19 (68%)	11 (40%)	30 (53%)	χ^2^(2) = 4.855, *p* = 0.072

**Table 2:** Descriptive statistics for DASS-21 subscales at pre-test and post-test outcomes.

	Psychoeducation Group *n* = 28		Waitlist Control Group *n* = 28	
DASS-21	Pre M (SD)	Post M (SD)	Pre M (SD)	Post M (SD)
Depression	6.00 (4.01)	4.96 (5.1)	6.39 (4.75)	7.39 (6.63)
Anxiety	5.43 (3.88)	4.68 (4.53)	4.89 (3.76)	5.57 (4.72)
Stress	7.89 (4.82)	5.86 (3.61)	7.68 (5.19)	8.54 (5.4)

**Table 3:** Intention-to-treat analysis of the DASS-21 subscales after multiple imputation (*N* = 154, *m* = 100 imputed datasets).

DASS-21	Psychoeducation M (SD)	Waitlist M (SD)	*b*	95% CI	*p*	*d*	FMI
Depression	6.17 (5.83)	7.34 (6.26)	−1.53	[−3.63, 0.57]	0.150	−0.25	0.61
Anxiety	5.59 (4.76)	5.91 (4.69)	−0.94	[−2.83, 0.94]	0.318	−0.20	0.67
Stress	6.49 (4.16)	8.57 (4.99)	−2.48	[−4.24, −0.72]	0.007	−0.53	0.62

Note: M (SD) = pooled post-intervention scores across imputed datasets; *b* = baseline-adjusted mean difference (psychoeducation − waitlist control), negative values favor the intervention; CI = confidence intervals; FMI = fraction of missing information.

## Data Availability

The datasets generated and/or analyzed during the current study are available from the corresponding author upon reasonable request.

## Supplementary Materials

The following supporting information can be downloaded at: https://www.mdpi.com/article/10.3390/nursrep16090295/s1, Table S1: Baseline characteristics of completers and non-completers; Table S2: Sensitivity analyses for the intention-to-treat analysis of the DASS-21 subscales.

## Public Involvement Statement

No public involvement in any aspect of this research.

## Guidelines and Standards Statement

This manuscript was drafted against the CONSORT guidelines [40] for RCT studies. The study was part of a large project funded by Austrian Research Promotion Agency, grant number 880557 (Care4Stress), that was preregistered at the German Clinical Trials Register (DRKS00025737), Date of Registration: 8 July 2021.

## Abbreviations

The following abbreviations are used in this manuscript:

DASS-21	Depression Anxiety Stress Scales [33]
COVID-19	Coronavirus disease of 2019
RCT	Randomized Controlled Trial
CHES	Computer-based Health Evaluation System
SPSS 26	Statistical Package for the Social Sciences
rMANOVA	Repeated measure Multivariate Analysis of Variance
MAR	Missing-at-random
