# Supplementary material for: Effects of a Seven-Week App-Based Passive Psychoeducation Program on Perceived Stress in Nursing Students—A Randomized Controlled Parallel-Group Study
Source: Nurs Rep. 2026 Aug 24;16(9):295. doi: 10.3390/nursrep16090295 (PMC13610340; doi:10.3390/nursrep16090295)
Supplement: Supplementary file 1 [file nursrep-16-00295-s001.zip › nursrep-4470981-supplementary.pdf]

Supplementary Material

Effects of a Seven-Week App-Based Passive Psychoeducation Program on Perceived Stress in Nursing Students - a randomized controlled parallel-group study

Table S1. Baseline characteristics of completers and non-completers

**Baseline variable**	**Completers (*n* = 56)**	**Non-completers (*n* = 98)**	***t***	***p***	***d***
Age (years)	28.64 (11.27)	27.11 (10.81)	0.83	.406	0.14
DASS-21 Depression	6.20 (4.36)	6.83 (4.76)	-0.81	.417	-0.14
DASS-21 Anxiety	5.16 (3.80)	6.23 (4.39)	-1.53	.128	-0.26
DASS-21 Stress	7.79 (4.96)	8.24 (4.34)	-0.60	.550	-0.10

Note. Values are *M* (*SD*). Completer status was defined by the availability of the post-intervention DASS-21 assessment. *d* = Cohen's *d* for independent samples; *df* = 152 for all comparisons. Dropout was equally distributed across arms (49 of 77, 63.6%, in each arm). Little's test of the assumption that data were missing completely at random was non-significant, $\chi^2(3) = 3.56$, $p = .313$. A logistic regression predicting dropout from group allocation, all baseline variables listed above, and sex was non-significant overall, $\chi^2(6) = 7.55$, $p = .273$; sex was the only individual predictor ($b = -0.92$, p = .036).

Table S2. Sensitivity analyses for the intention-to-treat analysis of the DASS-21 subscales.

**Approach**	***N***	**Depr. *b***	***p***	**Anx. *b***	***p***	**Stress *b***	***p***
Complete case	56	-2.04	.065	-1.34	.143	-2.82	.002
Multiple imputation (primary)	154	-1.53	.150	-0.94	.318	-2.48	.007
MI performed separately by arm	154	-2.01	.082	-0.90	.337	-2.55	.010
Baseline observation carried forward	154	-0.74	.062	-0.48	.149	-0.96	.010
Control-based ("copy reference")	154	-0.39	.656	-0.13	.863	-0.80	.315
δ-adjustment, +1 point	154	-0.90	.397	-0.32	.738	-1.85	.040
δ-adjustment, +2 points	154	-0.26	.804	+0.31	.741	-1.22	.176

Note. b = baseline-adjusted mean difference (psychoeducation - waitlist control) from an analysis of covariance with the corresponding baseline score as covariate; negative values favor the psychoeducation group. Complete-case *n* = 56 for all three subscales. Multiple imputation used chained equations with predictive mean matching, *m* = 100 imputed datasets; estimates were pooled using Rubin's rules. In the δ-adjusted pattern-mixture analyses, imputed post-intervention scores of dropouts in the psychoeducation group were shifted towards poorer outcomes by the stated number of scale

points. For the stress subscale, the tipping point at which the effect ceased to be significant was $\delta = 1.13$ points (approximately 0.25 *SD*).
